# Exploring Bacterial Cellulose and a Biosurfactant as Eco-Friendly Strategies for Addressing Pharmaceutical Contaminants

**DOI:** 10.3390/molecules29020448

**Published:** 2024-01-17

**Authors:** Nathália Roberta Cardoso Mendes Castanho, Nathane de Marco, Érika Leão Ajala Caetano, Patrícia Lius Melo Alves, Thaisa Borim Pickler, Natasha Lien de Almeida Ibanez, Angela Faustino Jozala, Denise Grotto

**Affiliations:** Department of Pharmacy, University of Sorocaba (UNISO), Sorocaba 18023-000, Brazil; nathalia.rcmc@gmail.com (N.R.C.M.C.); nathane.demarco@gmail.com (N.d.M.); erikacaetano01@hotmail.com (É.L.A.C.); 00105625@aluno.uniso.br (P.L.M.A.); thaisa.pickler@uniso.br (T.B.P.); natasha.lien@hotmail.com (N.L.d.A.I.)

**Keywords:** biosorption, bacterial cellulose, biosurfactant, hormones, micropollutants, drug

## Abstract

Aquatic environments face contamination by pharmaceuticals, prompting concerns due to their toxicity even at low concentrations. To combat this, we developed an ecologically sustainable biosurfactant derived from a microorganism and integrated it into bacterial cellulose (BC). This study aimed to evaluate BC’s efficacy, with and without the biosurfactant, as a sorbent for paracetamol and 17α-ethinylestradiol (EE2) in water. We cultivated BC membranes using *Gluconacetobacter xylinus* ATCC 53582 and synthesized the biosurfactant through pre-inoculation of *Bacillus subtilis* in a synthetic medium. Subsequently, BC membranes were immersed in the biosurfactant solution for incorporation. Experiments were conducted using contaminated water, analyzing paracetamol concentrations via spectrophotometry and EE2 levels through high-performance liquid chromatography. Results indicated BC’s superior adsorption for EE2 over paracetamol. Incorporating the biosurfactant reduced hormone adsorption but enhanced paracetamol sorption. Notably, original and freeze-dried BC exhibited better adsorption efficacy than biosurfactant-infused BC. In conclusion, BC showed promise in mitigating EE2 contamination, suggesting its potential for environmental remediation. Future research could focus on optimizing biosurfactant concentrations to enhance sorption capabilities without compromising BC’s inherent effectiveness.

## 1. Introduction

Human activities and rapid industrial advancement have heightened water pollution, placing significant focus on the monitoring of micropollutants in the environment. Compelling evidence highlights aquatic toxicity, endocrine disruption, induction of antimicrobial resistance, and other adverse effects [1,2]. This category of pollutants encompasses various pharmaceutical products from different classes, including analgesics, antibiotics, anti-inflammatories, and contraceptives. Additionally, it involves natural and synthetic hormones excreted by humans and other animals, such as estrone, estradiol, estriol, and ethinyl estradiol [3,4].

Currently, there is a lack of consensus or adequate evidence concerning safety limits required to prevent the adverse effects of these substances on the environment or human health. Consequently, pharmaceutical products, especially those with endocrine-disrupting characteristics like the hormones in contraceptives, have become a focal point of investigation [5]. Notably, 17α-estradiol (EE2) is a prominent estrogenic steroid capable of causing harmful effects (endocrine disruption) even at extremely low concentrations [6].

Another pharmaceutical compound that deserves attention is paracetamol, also known as acetaminophen, a potent analgesic and antipyretic both widely used over the counter and prescribed for pain and fever [7,8]. It is capable of inducing gastrointestinal, renal, and vascular side effects and liver damage [9,10]. However, toxicity is typically not acute, making it challenging to establish a clear link between the toxic agent and its long-term effects [11].

Sorption is the process by which a substance dissolved in a fluid permeates the solid portion of a porous medium. This phenomenon can transpire through absorption or adsorption [12]. Representing a highly effective method for recovering toxic compounds from aqueous solutions, various biomaterials, such as bacteria, yeast, fungi, and algae, have demonstrated success as biosorbents. Biosorption is an ecologically friendly approach used to remove metals [13,14]

Studies have demonstrated that the immobilization of microorganisms has been utilized as a biocatalyst in situations of environmental pollution [15,16,17]. However, research on the immobilization of bioproducts produced by microorganisms for the same purpose is limited. In recent years, considerable attention has been directed towards research on adsorption using surfactant mixtures. This interest arises from the intriguing interactions within these mixtures, resulting in remarkable interfacial effects, characterized by modifications in adsorption and surface charge density [18].

In recent years, the adsorption conducted by surfactant mixtures has garnered attention for creating a system with interactions between mixtures, resulting in pronounced interfacial effects marked by alterations in adsorption and surface charge density [19]. However, a biosurfactant has yet to be assessed for its immobilization in bacterial cellulose, particularly applied in the biosorption of pharmaceutical products. This study holds the promise of making substantial contributions to research in the field of environmental remediation and waste treatment. By delving into the utilization of BC and biosurfactants as environmentally friendly biosorbents for pharmaceutical contaminants, it introduces a fresh perspective in the development of sustainable and effective methods for pollutant removal in aquatic environments [20]. The findings and outcomes of this research may shed light on the efficiency and viability of these biosorbents in eliminating pharmaceutical contaminants from water sources. This could foster the advancement of eco-friendly techniques for wastewater treatment or environmental decontamination, thereby aiding in the preservation of aquatic ecosystems and public health. Furthermore, by highlighting the potential of BC and biosurfactants as eco-friendly alternatives for contaminant removal, this study might serve as a catalyst for future research endeavors. It could inspire the exploration of biodegradable materials and sustainable methodologies in waste and pollutant treatment, aiming to safeguard and conserve the environment.

Thus, a biosurfactant produced by a microorganism will be immobilized in bacterial cellulose, offering effective, economically viable, and environmentally friendly options as drug adsorbents.

## 2. Results

### 2.1. Biomaterials Characterization

**Figure 1:**
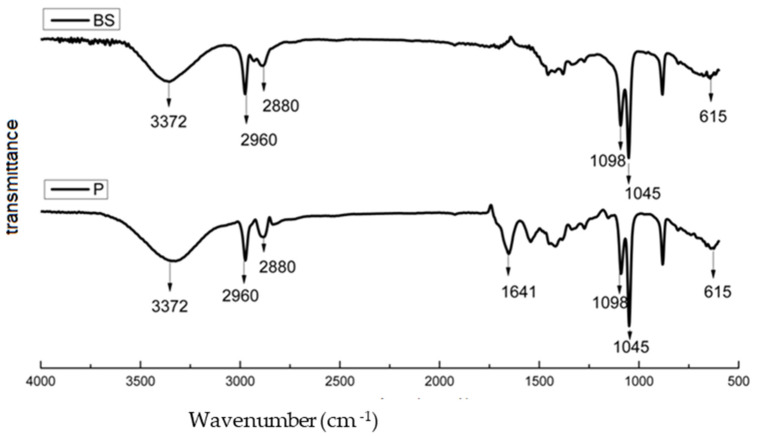
Fourier Transform Infrared Spectroscopy (FTIR) transmittance spectra of both the biosurfactant produced (BS), in freeze-dried powder form, and the surfactin pattern (P), used as a standard.

The comparison between the biosurfactant (BS) produced and standard surfactin is depicted in Figure 1, illustrating the characterization run by Fourier Transform Infrared Spectroscopy-FTIR, using transmittance modes. The bands were associated with the main chemical groups, compared to the standard. Notably, the bands appearing in the spectrum related to the resuspension of the lyophilized powder in methanol closely resemble those documented in the literature and align with the analyzed surfactin standard.

**Figure 2:**
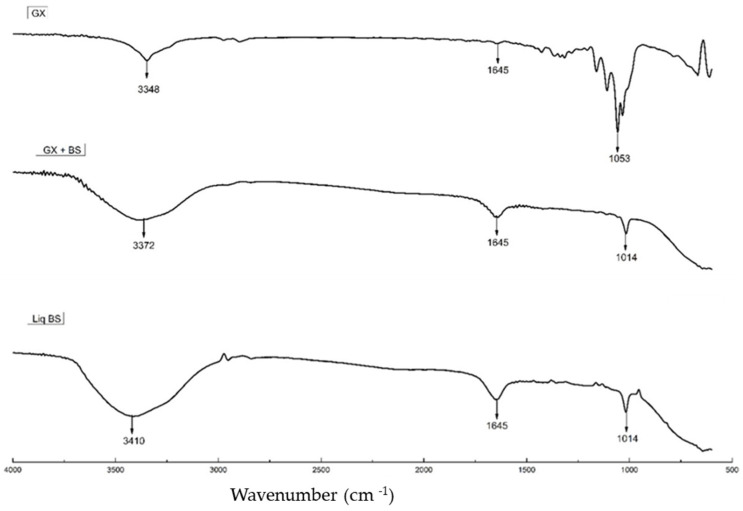
FTIR Transmittance Spectra of biosurfactant (BS) incorporation: ‘GX’ for bacterial cellulose from Gluconacetobacter xylinus, ‘GX + BS’ for bacterial cellulose with incorporated BS, for washed bacterial cellulose with incorporated BS, and ‘Liq BS’ for BS in solution.

The incorporation of the biosurfactant into BC was also scrutinized using FTIR. As shown in Figure 2, the peaks characterizing the biosurfactant (BS) incorporated into the cellulose membrane are present, even after the membrane underwent washing. The analysis of the solubilized BS similarly revealed the presence of these same bands.

### 2.2. Adsorption Kinetics

**Figure 3:**
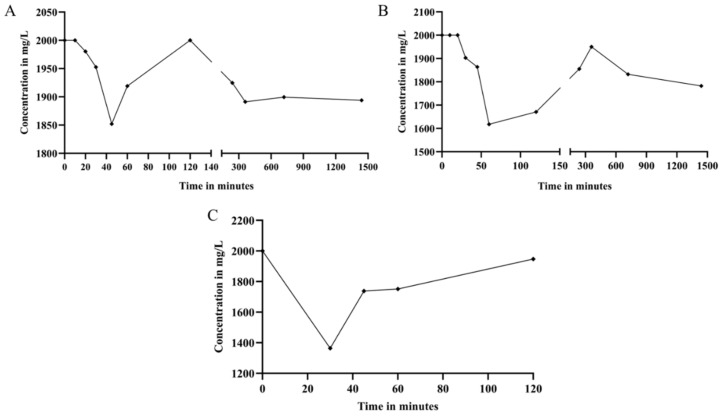
Paracetamol adsorption kinetics. In (**A**) paracetamol adsorption on bacterial cellulose; In (**B**) adsorption of paracetamol on bacterial cellulose + BS; In (**C**) adsorption of paracetamol on crushed and lyophilized bacterial cellulose without BS.

Figure 3 presents the analysis of paracetamol adsorption in three different samples. Figure 3A depicts the rate of paracetamol adsorption on bacterial cellulose, revealing the limited efficiency of the technique. Paracetamol remains in high concentrations in the medium, reaching its peak adsorption between 45 and 60 min, with an adsorption of 7.4% and 4.1%.

Figure 3B illustrates the kinetics of paracetamol adsorption on BC with the addition of BS, indicating intermediate performance of the technique. Paracetamol maintains significant concentrations in the medium, with peak adsorption occurring between 60 and 120 min, resulting in 19.1% and 16.5% adsorption.

For lyophilized and pulverized bacterial cellulose, a shorter contact time was chosen, as it had been observed that desorption occurred after 100 min. Figure 3C demonstrates the kinetics of paracetamol adsorption in this sample, showing good performance of the technique. Paracetamol still remains in high concentrations in the medium, reaching its peak adsorption between 30 and 45 min, with 31.8% and 13.11% of adsorption. An initial decrease in paracetamol concentration may be attributed to its rapid adsorption onto the material’s surface, indicating a strong initial affinity between paracetamol and the adsorbent. Subsequently, the rise in concentration suggests material saturation, wherein the initial adsorption capacity becomes filled, leading to an accumulation of paracetamol in the solution as the material reaches its maximum adsorption capacity.

**Figure 4:**
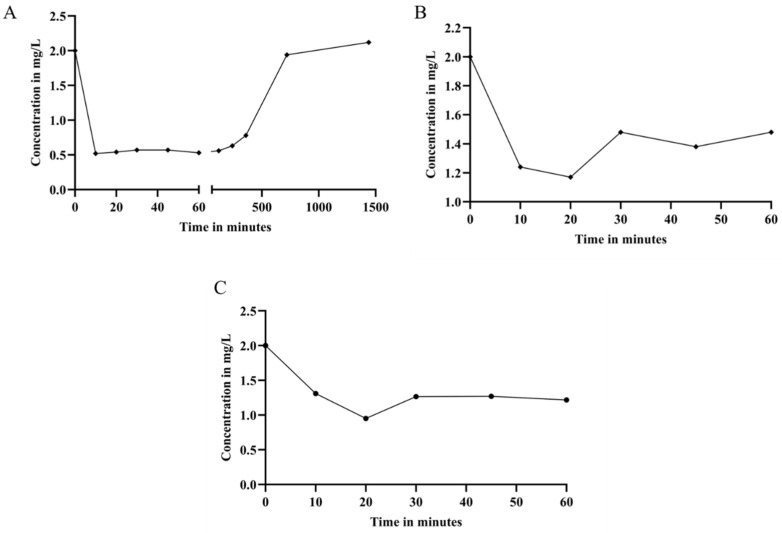
Adsorption kinetics of 17α-ethinylestradiol. In (**A**) adsorption of EE2 on bacterial cellulose; In (**B**) adsorption of EE2 on bacterial cellulose + BS; In (**C**) EE2 adsorption on crushed and lyophilized bacterial cellulose without BS.

Figure 4 displays the results of the analysis of EE2 adsorption on different BC samples. The evaluation was carried out using high-performance liquid chromatography (HPLC). The kinetic analysis of EE2 adsorption on bacterial cellulose, depicted in Figure 4A, highlights the remarkable effectiveness of this approach. The highest adsorption rate of EE2 occurs in the range of 10 to 60 min, with both points reaching an adsorption rate of 74%.

The adsorption kinetics of EE2 on BC in combination with the BS adsorbent, as illustrated in Figure 4B, shows a positive performance of this technique. The peak adsorption rate of EE2 is observed between 10 and 20 min, with adsorption levels reaching 38% and 42%, respectively. The analysis of the adsorption kinetics of EE2 on whole lyophilized bacterial cellulose, presented in Figure 4C, reveals an exceptional performance of this technique. The peak adsorption of EE2 occurs between 20 and 60 min, resulting in adsorption rates of 88% and 82%, respectively.

## 3. Discussion

Biosurfactants, a category of natural substances produced by microorganisms, have gained increasing recognition due to their significant role in various scientific disciplines and industrial sectors [21]. These surface-active agents of biological origin play a fundamental role in the solubilization of water-insoluble substances, reducing the surface tension of liquids and stabilizing emulsions [22].

Moreover, their impressive biodegradability and reduced ecological footprint, in contrast to conventional chemical surfactants, position biosurfactants as a hopeful substitute in a spectrum of applications, ranging from remediating pollutants to sectors such as petroleum, agriculture, food, cosmetics, and pharmaceuticals [23].

In our research, mirroring the approach of Vedaraman and Venkatesh (2011) [24], we encountered certain challenges during the laboratory-scale biosurfactant production process, which included pre-cultivation and cultivation in Erlenmeyer flasks. This procedure involved three sequential centrifugation steps: the first aimed at cell removal, while the second, similar to the method described by Das et al. (2008) [25], involved acid extraction to obtain a pre-purified biosurfactant. Lastly, the process featured a liquid–liquid extraction via centrifugation, followed by maintaining the precipitate until the complete evaporation of any solvents, culminating in freeze-drying.

As indicated in Figure 1, the peak bands between 3340 and 3365 cm^−1^ can be attributed to possible axial deformations of OH [26]. Furthermore, other bands observed in both the produced BS and the surfactin standard include the bands at 2973 cm^−1^ and 1045 cm^−1^, related to methyl CH stretching [27,28], with the band at 2883 cm^−1^ referring to CH2 [29] and the band at 1092 cm^−1^ referring to OC in the molecule [30].

The cellulose utilized in this study was obtained from the bacterium *Gluconacetobacter xylinus* (ATCC 53582), following the same approach as Jozala and colleagues [31]. BC is recognized as a highly effective polymer due to its inertness, high crystallinity, hydrophilicity, permeability, and remarkable mechanical resistance, as mentioned by Popa (2022) [32]. FTIR analysis, in line with Pinto’s (2013) [33] observations, reveals peaks characteristic of bacterial cellulose. This can be clearly observed in Figure 2, where the band at 3300 cm^−1^ represents the OH group, the band at 1600 cm^−1^ is attributed to the CH2 deformation, and the band at 1000 cm^−1^ is related to CO/CC.

After integrating the BS bioproduct, the kinetic tests typically applied in the biosorption of metals [13,14] were conducted. In our study, which focused on the drugs acetaminophen (paracetamol) and the hormone 17α-ethinylestradiol, the adsorption rates by the bioproduct in relation to these drugs were analyzed, considering the interaction of the mixtures and the interfacial effects generated. This includes the evaluation of parameters such as surface charge density and porosity, as highlighted by Ayoub (2021) [34].

Singh et al. (2007) [35] observed that both chemical and biological surfactants can have variable effects on the speed of pollutant bioremediation, without the ability to accurately predict the results, emphasizing the need for empirical confirmation. Pacwa-Płociniczak et al. (2011) [36] discussed the ability of biosurfactants to form micelles that can offer protection to contaminants, potentially inhibiting degradation. Furthermore, Guo et al. (2019) [37] demonstrated that the concentration of rhamnolipids, a type of biosurfactant, can influence the mobility and dissociation of the contaminant. This finding allows us to draw parallels with the rapid desorption observed in analyses involving BC containing biosurfactant. Table 1 shows other compounds and other contaminants, for comparative purposes.

According to Żółtowska-Aksamitowska et al. (2018) [38], the sorption capacity of chitin/lignin increases with the amount of solvent (paracetamol), being proportional to this amount. On the other hand, studies with BC showed different results, showing minimal adsorption over short periods of time. Ferandin Honorio et al. (2018) [39], when investigating the adsorption of 17β-estradiol on rice husk, observed that the ideal period for adsorption was 120 min. For 17α-ethinylestradiol, the best adsorption time using BC was 20 min. Additionally, Ferandin Honorio et al. (2018) [39], analyzed the adsorption process of the hormone 17β-estradiol by rice husk and soybean husk directly in pig manure. These animals receive hormonal supplementation.

Silva et al. (2018) [40] highlighted that cellulose modified with phthalic anhydride (used as an adsorption matrix) favors hydrogen bonds and electrostatic interactions with dyes, enabling the comparison of these interactions with BC in the context of EE2. On the other hand, Debs et al. (2019) [41], who studied biosorption by yeasts in the ethanol industry, also mentioned the hypothesis that sorption may increase due to electrostatic effects.

## 4. Materials and Methods

### 4.1. BC Production

From the strain *Gluconacetobacter xylinus* ATCC 53582, BC was cultivated in synthetic Hestrin & Schramm medium (20 g/L glucose, 5 g/L bacteriological peptone, 5 g/L yeast extract, 2.7 g/L Anhydrous sodium phosphate; 1.5 g/L citric acid monohydrate). Cultivation was performed in plates with 24 wells, each well containing 1 mL of inoculum with 10^6^ microorganisms. The plates were maintained for 7 days in static culture at 30 °C. After growth, the membranes were washed in running water and immersed in a 1 M NaOH solution under agitation at 60 °C for 1 h 30 min. Subsequently, the membranes were washed until reaching neutral or slightly acidic conditions (pH between 5.0 and 7.0), autoclaved at 121 °C for 15 min in MilliQ water, and stored at 4 °C, a technique adapted from Jozala et al. (2015) [31].

Following this procedure, a portion of the cellulose was crushed and stored in a biofreezer set at −80 °C (Thermo Scientific™REVCO^®^ ULT-1386-3-D, Waltham, MA, USA.) for approximately 24 h. Subsequently, the frozen samples underwent freeze-drying using a Thermo Savant Freeze Dryer LK-40 for about 48 h (Supplementary Material Figure S1).

### 4.2. Biosurfactant Production

The Bacillus subtilis ATCC 11,774 primary culture was performed utilizing 50 mL of the standard Tryptone Soy Broth (TSB) (Merck-Millipore^®^, São Paulo, SP, Brazil) culture media in 125 mL Erlenmeyer flasks. The flasks were kept in an orbital shaker (Nova Técnica, NT 715—Piracicaba, SP, Brazil) at 35 °C and 150 rpm for 96 h. The primary culture was filtered using a 0.25 µm particle retention filter (Merck-Millipore^®^, São Paulo, SP, Brazil) to evaluate the initial mass of the process. The metabolic viability of B. subtilis was measured by its ability to produce biosurfactant (BS). After 96 h of primary culture, the samples were centrifuged (Thermo Scientifc Centrifuge model ST16R, Waltham, MA, USA) at 4 °C, 5000 rpm for 30 min to obtain a cell-free supernatant. The supernatant was acidified to pH 2.0, utilizing 1 M hydrochloric acid (HCl), and it was kept overnight (±16 h) at 4 °C. After that, the acid sample was centrifuged (4 °C, 5000 rpm for 30 min) [42]. The supernatant was disposed, and the remaining pellet was resuspended in 3 mL Milli-Q water, mixed with 8 mL of chloroform and 4 mL of methanol. This solution was manually stirred five times and centrifuged at 25 °C, 5500 rpm for 10 min. A three-phase system was constructed, the interface phase was separated, and its solvents were evaporated [43]. After the evaporation, the sample was kept at −80 °C for 24 h, and it was lyophilized for 72 h. The BS production was measured by Fourier-Transform Infrared Spectroscopy (FTIR).

### 4.3. Incorporation

The resuspension involved reconstituting 0.1 g of freeze-dried biosurfactant in 10 milliliters of methanol to submerge both the bacterial cellulose and the crushed, freeze-dried bacterial cellulose. The test was conducted in 24-well plates, with each well containing 0.5 g cellulose and 1 mL of the biosurfactant solution. The plates were agitated at 25 °C at a speed of 100 rpm for a duration of 24 h.

All BC samples, with or without the added biosurfactant, and the biosurfactant resuspension were analyzed by Fourier Transform Infrared Spectroscopy (FTIR) in the wavelength range of 4000 to 500 cm^−1^.

### 4.4. Adsorption Kinetics

To evaluate the biosorbent capacity, 0.5 g samples of whole BC membranes without surface water, with and without BS, were added to 60 mL of 2 g/L paracetamol and 2 mg/L 17α-ethylestradiol (EE2) solutions (pH values ranging between 5.0 and 8.0). The systems were kept shaking, and samples were taken at points in time of 10, 20, 30, 45, 60, 120, 240, 360, 720, and 1440 min. Samples of whole, lyophilized, and pulverized cellulose without BS were taken at times of 10, 20, 30, 45, 60, and 120 min. The samples were filtered and then analyzed using either a UV-Visible Spectrophotometer or High Performance Liquid Chromatography.

### 4.5. Evaluation of Acetaminophen with a UV-Visible Spectrophotometer

Acetaminophen was analyzed by spectrophotometry, following a methodology adapted from Shihana et al. (2010) [44]. To achieve this, it was necessary to standardize the medication dosage, using a calibration curve from 0.1 to 10 g/L (represented by the equation y = 0.0233x − 0.0019, with a coefficient of determination (R^2^) of 1). Sodium nitrite (NaNO_2_ 10g%) and hydrochloric acid (HCl 6M) were added to the water samples, leading to nitration. In basic medium, with sodium hydroxide (NaOH 50%) and ammonium sulfate ((NH_4_)_2_SO_4_ 15%) added, the solution turned yellow (azo dye), with absorption at a wavelength of 430 nm. To treat the samples taken at the established times above, it was necessary to carry out deproteinization, using trichloroacetic acid (C_2_HCl_3_O_2_ 15%), followed by nitration, adding 10g% NaNO_2_ and 6 M HCl to the samples (forming the compound 2-nitro-5-acetaminophenol). After showing a slight yellow color, 15% (NH_4_)_2_SO_4_ and 50% NaOH were added, and the reading was taken on a spectrophotometer, at a wavelength of 430 nm.

### 4.6. Evaluation of Ethinylestradiol with High Performance Liquid Chromatography

EE2 analysis was performed using High Performance Liquid Chromatography (HPLC). For this, standardization of the medication dosage was necessary through a calibration curve, with concentrations from 0.025 to 2 mg/L, thus measuring the area of the peaks in the HPLC program. The data were adjusted to a linear equation represented by y = 115,131x − 420, and it presented a coefficient of determination (R^2^) of 0.9991.

The samples taken at the mentioned times were read on the Liquid Shimadzu chromatograph–Model Class-VP, using a C18 column 125 mm high, 4.60 mm in diameter and filled with 5 µm (Thermo Scientific) at 37 °C in the oven. The results were collected with a 20 µL injection and a run time of 6 min with detection at 202 nm. The mobile phase was composed of 70% HPLC standard acetonitrile (sigma-Aldrich) and 30% ultrapure water–Milli-Q in an isocratic system (Unruh, 2011). The equipment’s software gives us the peak area, in a chromatogram, and then the EE2 concentration is calculated from the straight-line equation.

### 4.7. Data Analysis

Data were expressed as absolute results or a percentage of sorption. The adsorption capacity (AC) of bioproducts and the percentage of removal (%*R*) were determined by the equations:

$$\frac{CA=\left(Co-Ce\right)\times V}{mpb}$$

and

$$\frac{\%R=\left(Co-Ce\right)\times 100}{Co}$$

where *Co* is the initial concentration, *Ce* is the concentration observed after contact with the bioproduct, *V* is the total volume of the solution, and mbp is the mass of the bioproduct. The results were analyzed using the software programs Oringin 8 and GraphPad Prima^®^.

## 5. Conclusions

Our findings affirm the successful production of the biosurfactant and its seamless integration into bacterial cellulose. Adsorption experiments demonstrated that freeze-dried BC exhibited the highest efficiency in adsorbing the contaminant EE2, while BC without surface water showed proficiency in paracetamol adsorption. The addition of the biosurfactant improved the adsorption of paracetamol but decreased the adsorption of EE2. In conclusion, it is plausible that bacterial cellulose, particularly in its freeze-dried state, holds promise as an environmentally friendly biosorbent for extracting certain pharmaceuticals from polluted waters.

## Figures and Tables

**Table 1:** Studies with different materials (sorbents or biosurfactants) for removal of organic contaminants.

Materials	Contaminant	Experimental Effects	References
Rhamnolipidic biosurfactants	17α-ethinylestradiol	The concentration of rhamnolipids, a type of biosurfactant, can influence the mobility and dissociation of the contaminant.	Guo et al. (2019) [37]
Chitin and lignin	Ibuprofen and acetaminophen	The sorption capacity of chitin/lignin increases with the amount of solvent	Żółtowska-Aksamitowska et al. (2018) [38]
Rice husk biomass	17 β-estradiol	The biosorbent displayed typical functional groups of cellulose, hemicellulose, lignin, and proteins, with an amorphous, fibrous, and porous surface	Ferandin Honorio et al. (2018) [39]
Phthalic anhydride-modified cellulose	Crystal Violet and Methylene Blue” dyes	Phthalic anhydride-modified cellulose promotes hydrogen bonding and electrostatic interactions with dyes	Silva et al. (2018) [40]
East biomass	17α-ethinylestradiol	Yeast biosorption in the ethanol industry, mentioning the hypothesis that sorption may increase due to electrostatic effects.	Debs et al. (2019) [41]

## Data Availability

Data are contained within the article and Supplementary Materials.

## Supplementary Materials

The following supporting information can be downloaded at: https://www.mdpi.com/article/10.3390/molecules29020448/s1. Figure S1. Crushed and lyophilized bacterial cellulose.
