# Supplementary material for: Exploring Bacterial Cellulose and a Biosurfactant as Eco-Friendly Strategies for Addressing Pharmaceutical Contaminants
Source: Molecules. 2024 Jan 17;29(2):448. doi: 10.3390/molecules29020448 (PMC10818349; doi:10.3390/molecules29020448)
Supplement: Supplementary file 1 [file molecules-29-00448-s001.zip › molecules-2767590-supplementary.pdf]

Supplementary Material

Exploring Bacterial Cellulose and a Biosurfactant as Eco-Friendly Strategies for Addressing Pharmaceutical Contaminants

Nathália Roberta Cardoso Mendes Castanho [1], Nathane de Marco, Érika Leão Ajala Caetano, Patrícia Lius Melo Alves, Thaisa Borim Pickler, Natasha Lien de Almeida Ibanez, Angela Faustino Jozala * and Denise Grotto *

Department of Pharmacy, University of Sorocaba (UNISO), Sorocaba 18023-000, Brazil; nathalia.rcmc@gmail.com (N.R.C.M.C.); nathane.demarco@gmail.com (N.d.M.); erikacaetano01@hotmail.com (É.L.A.C.); 00105625@aluno.uniso.br (P.L.M.A.); thaisa.pickler@uniso.br (T.B.P.); natasha.lien@hotmail.com (N.L.d.A.I.)
* Correspondence: angela.jozala@prof.uniso.br (A.F.J.); denise.grotto@prof.uniso.br (D.G.); Tel.: +55-15-2101-7104 (A.F.J. & D.G.); Fax: +55-15-2101-7000 (A.F.J. & D.G.)

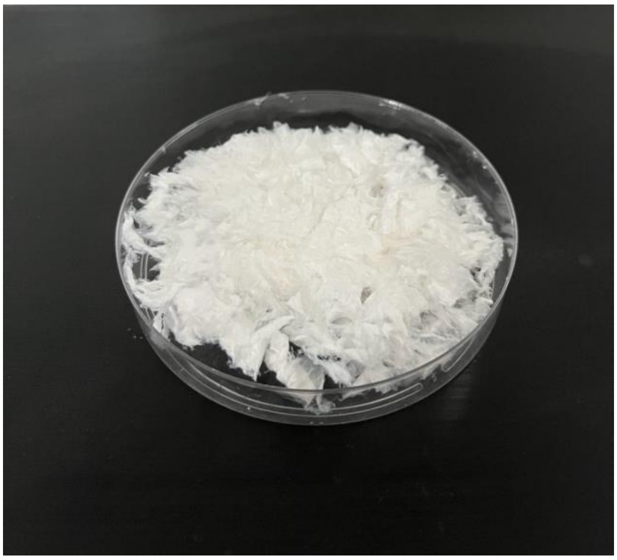

Figure S1. Crushed and lyophilized bacterial cellulose.
